# The International Neuromodulation Registry: An Informatics Framework Supporting Cohort Discovery and Analysis

**DOI:** 10.3389/fninf.2020.00036

**Published:** 2020-09-18

**Authors:** David M. Hedges, John C. Hegman, Jefferson R. Brown, Jack T. Wilburn, Brian E. Chapman, Christopher R. Butson

**Affiliations:** ^1^Scientific Computing and Imaging (SCI) Institute, The University of Utah, Salt Lake City, UT, United States; ^2^Department of Biomedical Informatics, The University of Utah, Salt Lake City, UT, United States; ^3^Department of Radiology and Imaging Sciences, The University of Utah, Salt Lake City, UT, United States; ^4^Department of Biomedical Engineering, The University of Utah, Salt Lake City, UT, United States; ^5^Departments of Neurology, Neurosurgery and Psychiatry, The University of Utah, Salt Lake City, UT, United States

**Keywords:** deep brain stimulation, responsive neurostimulation, transcranial magnetic stimulation, graph database, spinal cord stimulation

## Abstract

**Background:**

Neuromodulation therapies, such as deep brain stimulation (DBS), spinal cord stimulation (SCS), responsive neurostimulation (RNS), transcranial magnetic stimulation (TMS), transcranial direct stimulation (tDCS), and vagus nerve stimulation (VNS) are used to treat neurological and psychiatric conditions for patients who have failed to benefit from other treatment approaches. Although generally effective, seemingly similar cases often have very different levels of effectiveness. While there is ongoing interest in developing predictors, it can be difficult to aggregate the necessary data from limited cohorts of patients at individual treatment centers.

**Objective:**

In order to increase the predictive power in neuromodulation studies, we created an informatics platform called the International Neuromodulation Registry (INR). The INR platform has a data flow process that will allow researchers to pool data across multiple centers to enable population health research.

**Methods:**

This custom informatics platform has a Neo4j graph database and includes a harmonization process that allows data from different studies to be aggregated and compared. Users of the INR can download deidentified patient imaging, patient demographic data, device settings, and medical rating scales. The INR supports complex network analysis and patient timeline visualization.

**Results:**

The INR currently houses and allows visualization of deidentified imaging and clinical data from hundreds of patients with a wide range of diagnoses and neuromodulation therapies.

**Conclusion:**

Ultimately, we believe that widespread adoption of the INR platform will improve population health research in neuromodulation therapy.

## Introduction

Neuromodulation is a broad class of therapies that use implanted or non-invasive electromagnetic stimulation to improve quality of life for patients with a wide range of neurological or psychiatric conditions (Sakas et al., 2007). These therapies generally meet the following four criteria (Holsheimer, 2003):

1. The therapy is dynamic and ongoing – not a short, non-recurring procedure.
2. Specific neural network activation is affected by ongoing electrical or magnetic stimulation of parts of its afferents, or by ongoing neuropharmacological stimulation affecting neurotransmission.
3. Clinical effects are continuously controllable by varying one or more stimulation parameters.
4. Therapy is non-destructive, and its physiological effect are reversible.

Common types of neuromodulation therapy include deep brain stimulation (DBS), spinal cord stimulation (SCS), responsive neurostimulation (RNS), transcranial magnetic stimulation (TMS), transcranial direct stimulation (tDCS), and vagus nerve stimulation (VNS). These therapies have been used to treat Parkinson’s disease, essential tremor, Tourette syndrome, traumatic brain injury, chronic pain, epilepsy, and several other neurological and psychiatric conditions. For some patients, neuromodulation therapy can provide dramatic symptomatic improvement, particularly when the system is tuned to suit the specific needs of the patient while avoiding intolerable side effects. Although many of these therapies have been shown to be effective in randomized, controlled trials, they are generally used only when less invasive methods prove ineffective for symptom management. However, even after rigorous screening and selection, and despite apparent similarity to other patients who respond well, some patients fail to receive adequate therapeutic improvement. Efforts to improve lead localizations using medical-imaging-based computational methods are underway, but so far have mixed results when correlating lead localization with medical outcomes (Starr et al., 2002; Nestor et al., 2014; Koivu et al., 2018).

Neuromodulation therapies can be incredibly powerful in treating several neurological conditions, but the therapeutic mechanisms are unknown, making it difficult to optimize patient care. This challenge is exacerbated as some patients may respond well to treatment whereas other patients may not, despite having similar preoperative conditions. The gold standard for assessing therapeutic efficacy is level one evidence resulting from randomized, blinded trials. These types of studies have been successful in some cases and unsuccessful in others, but either way are very expensive to conduct because of the surgical costs and long term follow required. In most cases these studies are conducted by companies who are testing a therapy prior to applying for market approval. In order to avoid biases from smaller studies (Schlaepfer and Fins, 2010), problems such as these necessitate the use of large-scale population studies with accompanying complex statistical models to parse out small details that otherwise remain obscure. However, obtaining large sample sizes is difficult: clinical neuromodulation is relatively rare and individual medical centers deal with small numbers of patients, which means that individual researchers are hampered in their efforts to improve neuromodulation by chronically underpowered studies (Schlaepfer and Fins, 2010). Researchers addressing this problem by creating databases where data can be aggregated; however, no single registry or database exists that allows quantitative comparison of outcomes across neuromodulation methods and indications. Existing databases are largely limited in focus to a single disease [e.g., the International Tourette Syndrome Deep Brain Stimulation Public Database and Registry (Martinez-Ramirez et al., 2018)] or neuromodulation modality [e.g., the Aarhus Neuromodulation Database for SCS (Meier et al., 2013)]. Since many neuromodulation therapies have common feature, there has been a call for a single, query-able system encompassing all types of neuromodulation (Synofzik et al., 2012).

To address this need, we have created the International Neuromodulation Registry (INR), which admits data from all types of neuromodulation therapies and related indications. The registry is built using a Neo4j graphical database backend that is coupled with a web-based interface to allow for efficient data retrieval, cohort discovery, and analyses. Additionally, the INR welcomes independent researchers to both contribute data and perform their own analysis and cohort discovery. We are committed to maintaining a low-cost, functional, and simple system to allow researchers and clinicians alike to not only improve neuromodulation therapies, but provide a platform for better understanding the physical mechanisms underlying the effectiveness of neuromodulation.

## Materials and Methods

### Overview

The INR is a database with a web-based frontend at the following URL: https://neuromodulationregistry.org/. The landing page contains details about the user account and has links to the database functionality: Explore, Imaging, Account, Datasets, Upload, Learn. Currently, the INR has several functions on the landing page to facilitate cohort discovery and search; users are also able to extract deidentified medical imaging and patient-specific data. The “Explore” feature is powered by the Neo4j data browser. This powerful tool visualizes complex data relationships using node link diagrams. A working knowledge of the Neo4j database query language, Cypher, is necessary to run more complex queries than are possible using the node link diagram alone. Example Cypher queries and links to interactive Neo4j/Cypher tutorials are provided. Additionally, a new push toward an ISO standard graph query language, GQL, is being spearheaded by Neo4j and other graph database vendors. Since GQL is being developed in cooperation by Neo4j, we expect this new standard will be functional in our graph database instance starting in 2021, the proposed delivery date (Neo4j, 2020). We anticipate that this feature of the INR, which connects seemingly disparate datasets using network analysis techniques, will complement the cohort discovery feature and lead to scientific discovery by giving researchers and physicians valuable insights. Links are also provided to pages detailing the various datasets that are available (“Data Sets”) and links to the INR publication policy and data use agreement.

The Neo4j database, XNAT database, Django framework and web interface are integrated using Python^1^. Python is a versatile language with strong community support. The power of Python comes largely from extensive libraries and application programming interfaces (APIs). Python is used to power the frontend, query the backend, and provide updates for the database, giving us the flexibility to add features from existing Python libraries. This continuity facilitates future additions to and features of the INR, including the potential for machine learning analyses of neuromodulation data and the potential for a future API.

### Graph Databases

The relational database model (SQL) has been widely successful and almost universally implemented since its creation in the early 1970s. However, relational databases require a rigid data schema that largely prohibits the flexibility of adding data elements after the creation and implementation of the initial data model. Importantly, relationships are defined only as the data are retrieved through queries, often through computationally expensive “join” operations. Conversely, the data model in a graph database is incredibly flexible and allows for the addition of new data elements after the initial deployment. Data relationships are defined as the database is constructed and the data model evolves, enabling much faster data retrieval as the database grows. Unlike SQL databases, it is also possible to define attributes for a single patient without creating a sparse table, allowing memory conservation. A survey of the database management systems (DBMS) MySQL and Neo4j found that Neo4j was up to 10 times faster than MySQL (Vicknair et al., 2010). Graph databases also have tools for visually exploring the data and analyzing complex data relationships. Finally, a key advantage of a graph database over a relational database is the ability of a graph database to handle timeline events: a graph database provides the ability to visualize, store, and extract timeline-specific data, whereas temporal queries can be difficult using SQL databases (Snodgrass, 1987; Krause et al., 2016; Sen et al., 2017).

We anticipate that the requirements for the registry and underlying database will evolve as neuromodulation therapies grow more advanced: for example, approval of neuromodulation therapies for new conditions and diseases, the development of new DBS hardware, or the expansion of TMS therapies. Thus, a flexible data model is high priority. Additionally, two important considerations for the database backend are the ability to handle patient timelines and the flexibility to visually explore the datasets to identify complex relationships in the data. To meet these requirements the INR was created using a graph.

Neo4j is a widely used and well-supported graph database with an active online community offering support and serves as the graph database backend of the INR. Neo4j developers have created numerous open-source drivers and interfaces in Python, including interfaces that are compatible with the Django web development platform. Importantly, Neo4j has an interface that allows the INR to be powered and connected to using Python, and is packaged with a powerful and sophisticated (but proprietary) data browser that allows for data exploration.

### Imaging

One key area critical to neuromodulation research is neuroimaging. The INR is built using a seamless interface with the open-source XNAT imaging storage database (Marcus et al., 2007). This interface is powered through queries utilizing the representational state transfer (REST) API in XNAT for retrieval through the INR webpage. Image volumes are coregistered using Advanced Normalization Tools (ANTS^2^) and stored as NIFTI volumes.

The INR is housed in the HIPAA-compliant Protected Environment (PE) at The University of Utah Center for High Performance Computing (CHPC^3^). DICOM image stacks are retrieved from internal and external sources and passed through an Orthanc server for de-identification^4^. This pipeline is developed specifically to scrub PHI from DICOM tags. Additionally, all dates are randomly offset for all patients to obscure PHI, but temporal relationships are internally consistent and accurate within each patient dataset.

### Data Model and Organization

**FIGURE 1:**
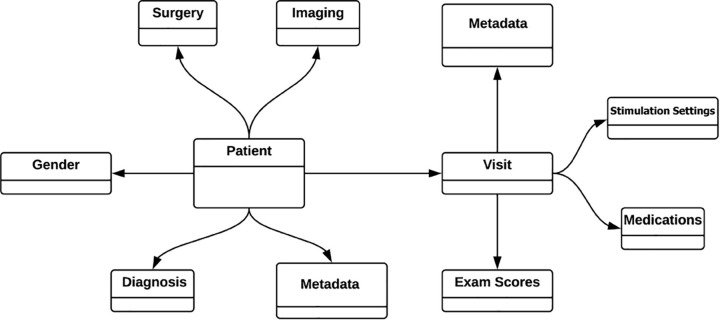
Simplified INR data model. All the data is modeled around and tied directly to each individual patient. Patient visits to the clinician (“Visit”) is a growing list of visits, but only shown here as a single visit for simplicity. One strength of the graph database is that all information is entered according to relationships, eliminating the need for “join” operations between tabulated information.

To facilitate future research the INR is built on an extremely flexible data model that is structured around individual patients. Each patient is linked to demographic information (e.g., gender), information about surgically implanted devices, diagnosis information, imaging, and an expandable list of visits complete with programming settings, medications, and medical examination results (Figure 1).

### Data Sharing and Security

Three levels of users have access to the database (Table 1), all of whom need to have access approval granted by the database administrators following an access request. The lowest level of access is standard access, which gives users read-only permissions to the publicly available data. The next level of access is sensitive user access, which is granted for individual datasets. Thus smaller, more easily identifiable data subsets can be protected while limiting the risk of exposure. The final level of access is administrator access. Administrator access affords super user access to the database, including permission to upload data directly from the data quarantine center to the live database.

**TABLE 1:** Access and permission rights for users of the INR. Access type will be approved by the database administration team and will be determined based on use case and contributions.

**Access Type**	**Upload Privileges**	**Browse + Download Privileges**
Standard Access	Quarantined	Standard
Sensitive Access	Quarantined	Standard + Conditional*
Admin Access	Full	All

*Access to data on sensitive populations will be determined on a case-by-case basis and will be limited to certain datasets.

Datasets in the INR can be given two flags that limit public access: sensitive and embargoed. Although all data housed in the INR is deidentified of protected health information, some of the datasets will be flagged sensitive if they represent individuals in such a sufficiently small experimental population that it is theoretically possible for the public to identify them, for example with the aid of social media. Several metrics are used to determine which datasets will be classified as sensitive, including sample size, rarity of the treatment, and the FDA approval status of the treatment. Sensitive datasets are described but are not accessible to standard users; access will be granted only after review of an individual request. Embargoed datasets are those that contributors desire to limit access to for a requested length of time either for their labs to retain publication priority or for other legal requirements. After the embargo expires, the data will become public. If contributors do not request an embargo, the data will be immediately accessible following data import processing.

**FIGURE 2:**
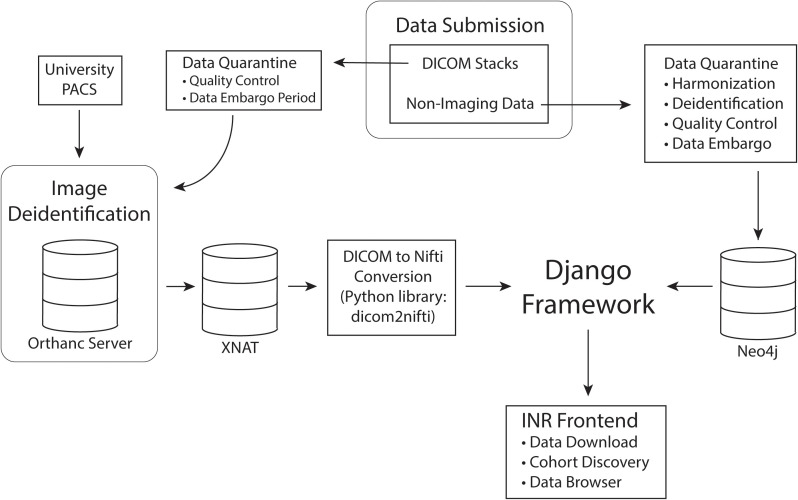
Data processing pipeline. Data is first submitted through the INR website and separated into imaging and non-imaging data. All data is quarantined for quality control and harmonization. If the contributing investigators wish to embargo their data for primary publication rights, the data will remain in quarantine for the length of the embargo period. While in quarantine, data will be thoroughly deidentified. After quarantine, imaging data will be permanently stored in XNAT and non-imaging data in Neo4j. Finally, the Django framework will connect the data to the INR frontend for queries and data exploration.

To preserve data integrity and prevent redundancy, users are currently unable to directly upload data to the INR. Users with standard and sensitive access will instead submit to a database administrator for preprocessing, harmonization and quality control. During preprocessing data is checked to ensure that it is deidentified and that the imaging meets basic quality control requirements. Once these requirements are satisfied, database administrators will notify the contributor and upload the data to the INR via a process that harmonizes the data with the INR data model. At this time, the contributor will be given standard user rights of access. After the quarantine period, the data will be rigorously scrubbed of PHI. The imaging is stored in XNAT while all other data is stored in Neo4j. Data will finally be tied into the central Django framework and made available on the INR frontend as diagrammed in Figure 2.

### Data Governance and Publication Rights

One challenge with data sharing is ensuring credit is given to those who provide the dataset(s) used by others. We use the Force11 policy on data citation, which states that “reproducible scholarship rests upon a foundation of robust, accessible data” and that datasets must be considered to be “citable products of research” (Martone, 2014). In accordance with this policy, when researchers use a dataset, they are obligated to cite the dataset in accordance with the Joint Declaration of Data Citation Principles: [dataset] authors; year; dataset title; data repository or archive; version (if any); persistent identifier (e.g., DOI). Specific details about the publications policy and data use agreement are available on the INR website at https://neuromodulationregistry.org/publication-policy/and https://neuromodulationregistry.org/data-agreement/. These agreements are adapted from similar agreements created by the Parkinson’s Progression Markers Initiative (PPMI^5^) and the Alzheimer’s Disease Neuroimaging Initiative (ADNI^6^). INR users also can request that datasets be coupled with an article citation. In this instance, when other researchers use such a dataset, they are required to cite both the dataset in the INR, but also the article supplied by the submitting researchers. A link to this paper will be available on the “Data Sets” page^7^.

## Results

### Patient Data in the Registry

The INR platform currently houses data from four datasets containing a total of 340 patients. These datasets include patients who have undergone DBS or RNS surgery in several countries. Patient diagnoses include Parkinson’s disease, Tourette syndrome, essential tremor, dystonia, and epilepsy. Stimulation targets include the subthalamic nucleus, ventral intermediate nucleus of the thalamus, centromedian nucleus of the thalamus, centrolateral nucleus of the thalamus, anterior limb of the internal capsule, or globus pallidus internus. The INR hosts demographic data, medical rating scales over sequential clinic visits, device programming parameters, and medical imaging (pre- and post-operative) for most patients. Currently, all patients in the INR have undergone either DBS or RNS surgery, but due to the flexible graph database framework, the database is capable of accommodating any form of neuromodulation data, including other surgical interventions such as VNS or non-invasive techniques such as TMS.

### Visualizations

**FIGURE 3:**
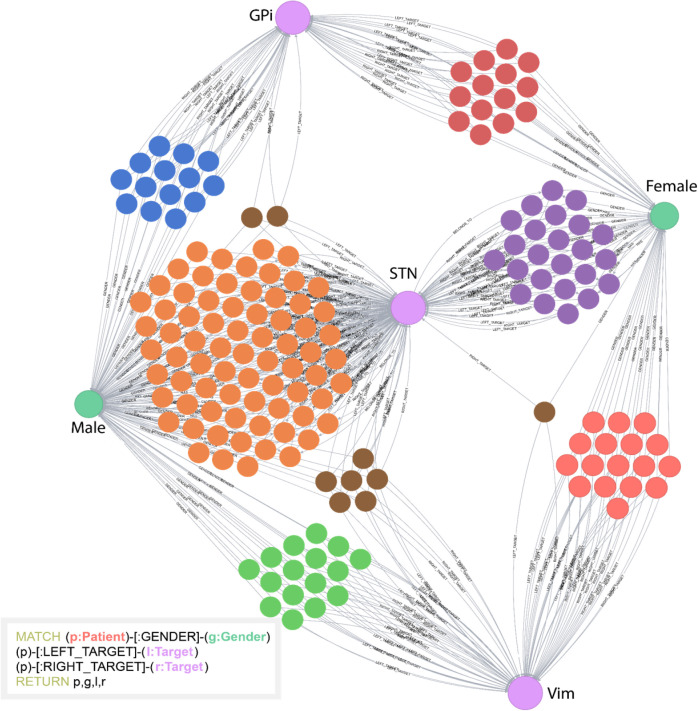
Example of a Cypher query and the visual result. In this example, the database was queried to return patients (unlabeled, multicolored nodes), gender (Male or Female), and stimulation targets (GPi, STN, or Vim). All unlabeled nodes represent individual patients. This simple example highlights how visual clustering analysis can easily be done using Cypher. The individual patient clusters share commonalities between the attributes selected. This helps users determine underlying structure of the data.

The INR platform supports complex network visualizations by utilizing the Neo4j Graph Browser. The browser allows users to both query and to visualize the data using a javascript visualization interface. This feature helps users better understand the timelines of patients, their clinical outcomes, and the factors that lead to these outcomes. An example of a Cypher query along with the visualization of the result is available in Figure 3.

**FIGURE 4:**
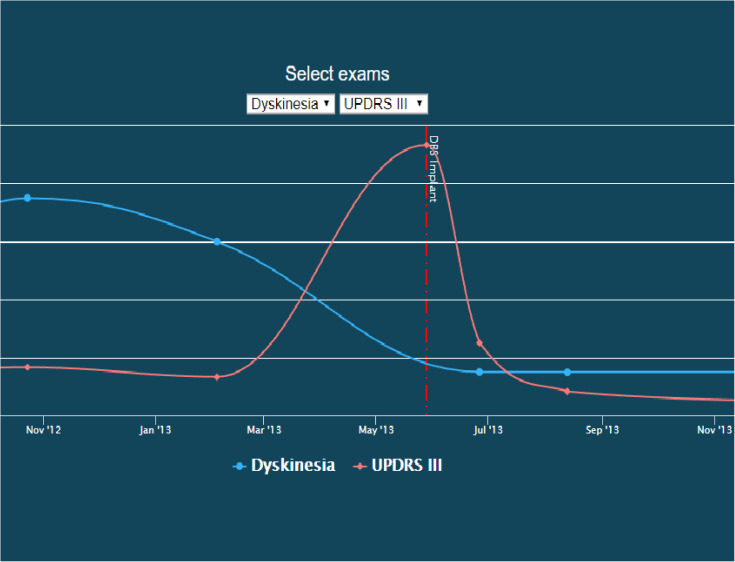
Screenshot of a patient timeline. In this image, dyskinesia exam scores are co-plotted with UDPRS III scores over time, illustrating their temporal relationship with the start of DBS therapy. This image is available on the INR website as an interactive explorer, where the user can control what is being plotted over time.

In addition to visualizing networks, we have developed a patient timeline visualization tool. Importantly, this tool does not require writing a database query; the visualizations are provided under the “Chart” tab for each patient record. Users may select two variables to simultaneously plot and compare. An example of this visual comparison is available in Figure 4.

## Discussion and Conclusion

### Need for Data Sharing and Integration

In 1979, the Belmont Report was announced and adopted as the United States’ guide to ethical human subjects research (National Commission for the Protection of Human Subjects of Biomedical and Behavioral Research, 1979). This report clearly states that human subjects research must maximize the potential benefit of the study. One simple way to meet this requirement is to allow data to be reanalyzed and reused. The INR provides a platform whereon neuromodulation data from center studies or clinical trials can be reanalyzed and recycled in future studies, thereby maximizing the potential benefit of the data.

### Data Harmonization

The intent of this informatics platform is to increase the predictive power for patients undergoing neuromodulation therapy. The INR platform is designed to combine datasets from different institutions to increase sample size. Building the backend on a graph database has given us the flexibility to ensure that all incoming datasets can be harmonized with our central data model. We are taking steps to ensure that all datasets are harmonized and that query results are directly comparable and unified behind a consistent naming scheme, which means that querying the database for cohort discovery will result in the ability to draw conclusions across different datasets.

### Future and Planned Developments

While the INR stores neuromodulation data in a next-generation graph database and supports both cohort discovery across different datasets and complex network analyses, we anticipate building an additional analytics layer into the framework of the INR. We are actively planning to include machine learning algorithms to support patient clustering and classification, with the intent to provide a clinical decision support tool for predicting both patient outcomes and stimulation parameters. In addition, the Butson lab has developed a suite of computational tools for processing neuroimaging and computing the volume of tissue activated, which can then be visualized via an iPad app. We anticipate that these computational tools can be leveraged by medical caregivers to streamline and optimize neuromodulation therapy to maximize patient quality of life.

### Outcomes of the INR

Fundamentally, researchers cannot create large-scale predictive algorithms from small patient cohorts and small datasets. This type of effective prediction can be done only by using large sample sizes. Larger datasets will lead to better predictive models. As data is combined from clinical trials (successful or failed) and from center studies into a harmonized and computable data model, better understanding of the processes and mechanisms can be generated, leading to enhanced understanding and the development of new technologies. A centralized database supporting this type of platform is essential to begin to exploit machine learning algorithms and other advanced computer science methodologies that are currently being explored in healthcare.

## Data Availability Statement

The raw data supporting the conclusions of this manuscript will be made available by the authors, without undue reservation, to any qualified researcher.

## Author Contributions

CB conceived the idea, carried out the experiment, and supervised the project. DH and JH constructed the conceptual design and implemented the physical design with help from BC, JB, and JW. DH wrote the manuscript with support from CB, JH, BC, JB, and JW. All authors contributed to the article and approved the submitted version.

## Conflict of Interest

CB has served as a consultant for NeuroPace, Advanced Bionics, Boston Scientific, Intelect Medical, Abbott (St. Jude Medical), and Functional Neuromodulation. CB is also a shareholder of Intelect Medical and is an inventor of several patents related to neuromodulation therapy. The remaining authors declare that the research was conducted in the absence of any commercial or financial relationships that could be construed as a potential conflict of interest.

## Footnotes

1 https://www.python.org/

2 https://stnava.github.io/ANTs

3 https://www.chpc.utah.edu/resources/ProtectedEnvironment.php

4 https://www.orthanc-server.com

5 https://www.ppmi-info.org/documents/ppmi-data-use-agreement.pdf

6 http://adni.loni.usc.edu/wp-content/uploads/how_to_apply/ADNI_DSP_Policy.pdf

7 https://neuromodulationregistry.org/data-sets/

## Abbreviations

CHPC: Center for High Performance Computing
DBMS: database management system
DBS: Deep brain stimulation
INR: International Neuromodulation Registry
PHI: protected health information
tDCS: transcranial direct current stimulation
TMS: transcranial magnetic stimulation
VNS: vagus nerve stimulation.
